# Loot

**Published:** 1872-06

**Authors:** 


### Loot.

### *Treatment of Cancrum Oris.*

Dr. McGreevy (*British Med. Journal*) says: "Of all the local remedies or applications I have resorted to in such cases, I have never found any application so useful or so effective as hydrochloric acid. Neither nitric acid, nitrate of silver nor chlorate of potash, nor any other remedy that I have ever tried or used, except hydrochloric acid, did I ever find to be of the least use to check cancrum oris. I have almost never found hydrochloric acid fail to check the progress of this dreadful disease at once, and bring on a most rapid and healthy action in the part. Nor does it cause so much pain or suffering to the little patient as one would suppose, seeing that the gangrenous spot is almost entirely without feeling at this time. This acid is easily applied to the ulcer by means of a feather or small camel-hair brush. I have cured many cases of cancrum oris by this means.—*Medical Archives.*

### *Heat as a Poison.*

It appears from some curious experiments of M. Claude Bernard, that heat, when it attains too high a degree, acts on animals like a poison, and destroys feeling and motion. The precise action of heat on the blood, according to M. C. B., is as follows: the blood of an animal killed by heat becomes black, the oxygen it contains is rapidly transformed into carbonic acid, and finally disappears. This is not a true toxical action, but rather an excitement of the vital and normal properties of the red particles. The black blood of the rabbit killed by heat is still living; it absorbs oxygen by contact with the air, and again becomes ruddy, if the experiment is tried in time. Between 167° and 190° F., however, the blood coagulates, loses its vital properties, and cannot again become red. Heat above a certain degree kills the muscles without killing the blood. The chemical character of this poisoning of the muscles by heat is the most obscure part of the subject. It now remains for chemists to analyze the phenomena which accompany the muscular rigidity and cessation of motion produced by heat, and thus to solve the problem of the precise action of this poison, as they have done in the case of certain others.—*Philadelphia Med. News.*

### *Injection of Ammonia as an Antidote to Chloroform Poison.*

The efficacy of this method has been tried recently in Melbourne, with very encouraging results. A person, while under the influence of liquor, drank an ounce of chloroform in two doses, and became insensible almost immediately. An emetic of salt and water, which was poured down his throat without delay, caused him to vomit. The injection of liquor ammonia into the veins was decided upon. Half a drachm of liquid ammonia was injected into a vein in the arm, the strength of the injection being one part of ammonia to two parts of water. The good effect upon the pulse was rapid and marked, and, after an interval of twenty-five minutes, another half-drachm was injected. The improvement increasing, a third injection was made, after another interval of twenty-five minutes. After three-quarters of an hour, another half-drachm of ammonia was injected, bringing the total amount of *antidote* introduced into the blood to two drachms. The deceased regained consciousness about midnight, and, with the sanction of his medical attendant, was removed, between seven and eight next morning, from a house of ill repute, where he had been staying, to his own residence. The deceased subsequently complained of weakness, and expired twenty-four hours afterwards. —*Medical Press and Circular.*

### *Myrrh in Dyspepsia.*

Dr. Delioux de Savignac, (*Bull. de Therap.*, Dec. 15, 1871) writing on the therapeutic properties of myrrh, observes: "The stomach is the portion of the digestive apparatus which has appeared to me to be most happily influenced by myrrh. My experience fully confirms the reality of the stomachic properties which have been attributed to it by the older writers. I have seen painful forms of dyspepsia rapidly relieved, and even cured, by the use of this gum-resin; sometimes I have given it in conjunction with other remedies, such as bismuth, magnesia, bicarbonate of soda, etc., drugs which had hitherto proved ineffectual, and sometimes I have employed it alone, so as to leave no doubt as to the efficacy of its action."—*Birmingham Medical Review*, April, 1872.

### *Ivy Poisoning.*

Mr. H. Markham, of Port Jefferson, New York, sends the following note to the *Scientific American:*

"I send you a prescription which, I am satisfied, from ten years' experience, is the very best remedy for ivy poisoning. It is simply to bathe the parts affected freely with *spirit of nitre*. If the blisters be broken, so as to allow the nitre to penetrate the cuticle, more than a single application is rarely necessary, and even where it is only applied to the surface of the skin three or four times during the day, there is rarely a trace of the poison left the next morning. Having often, previous to the discovery of this antidote, been rendered helpless and blind by ivy-poison, I know its worth to those affected thereby."—*Druggists' Circular*.

### *Boast Not.*

The late Mr. Lynn, an English surgeon, cut twenty-five patients for stone without losing one. He then boasted that he had, at last, discovered the secret of performing lithotomy with success. Afterwards he declared that the Almighty punished him for his presumption, for he lost the next four patients that he cut. —*Pacific Med. and Surg. Journal.*

It was rather a small business for the great God of the universe to kill four innocent afflicted men to dampen the feathers of a self-conceited, egotistical, surgical ass. This combination of egotism and blasphemy is a bow-shot beyond anything we have ever seen in print, or out of it. His last boast was greater than his first. Punished *him* by killing four other men!!!—*Nashville Jour. Med. and Surg.*

